# Is Olfactory Impairment Associated With 10-year Mortality Mediating by Neurodegenerative Diseases in Older Adults? The Four-Way Decomposition Analysis

**DOI:** 10.3389/fpubh.2021.771584

**Published:** 2021-11-26

**Authors:** Yang Cao, Zhenxu Xiao, Wanqing Wu, Qianhua Zhao, Ding Ding

**Affiliations:** ^1^Clinical Epidemiology and Biostatistics, School of Medical Sciences, Örebro University, Örebro, Sweden; ^2^Unit of Integrative Epidemiology, Institute of Environmental Medicine, Karolinska Institute, Stockholm, Sweden; ^3^Institute of Neurology, Huashan Hospital, Fudan University, Shanghai, China; ^4^National Clinical Research Center for Aging and Medicine, Huashan Hospital, Fudan University, Shanghai, China; ^5^National Center for Neurological Disorders, Shanghai, China

**Keywords:** olfactory, neurodegenerative disease, mortality, prospective, elderly

## Abstract

**Background:** Literature shows that olfactory impairment (OI) is associated not only with neurodegenerative diseases (NDDs), but also with increased mortality. In this study, we analyzed data collected from the prospective phase of the 10-year follow-up of the Shanghai Aging Study (SAS) to explore the mediation effect of NDDs on the OI-mortality relationship.

**Methods:** We analyzed data collected from the prospective phase of the 10-year follow-up of the SAS. We included 1,811 participants aged 60 years or older who completed both an olfactory identification test and a cognitive assessment at baseline (2010–2011). Survival status of the participants from baseline to December 31, 2019 was obtained from the local mortality surveillance system. We used the four-way decomposition method to attribute effects to interaction and mediation and to explore the mediation effect of NDDs on the OI-mortality relationship.

**Results:** The four-way decomposition method revealed a statistically significant association of OI with death. Overall, 43% higher risk for death was associated with OI [excess relative risk (ERR) = 0.43, 95% CI: 0.06–0.80, *p* = 0.023]. Excluding the mediation from NDDs and interaction between OI and NDDs, the controlled direct effect of OI on death was even higher in NDDs participants, with an ERR of 77% (95% CI: 0.00–1.55, *p* = 0.050). Statistically significant association was found for failure to identify coffee (ERR = 0.77, 95% CI: 0.18–1.36, *p* = 0.010) and marginally significant associations were found for failure to identify cinnamon (ERR = 0.33, 95% CI: −0.02–0.68, *p* = 0.068) and rose (ERR = 0.33, 95% CI: −0.01–0.67, *p* = 0.054) with death.

**Conclusion:** OI was associated with the long-term mortality in older adults and the association was even stronger in those with NDDs. Failure to identify coffee or rose was associated with a higher mortality risk, and the association was mediated by NDDs.

## Introduction

About 50–70% of people aged 65 years or older exhibit olfactory impairment (OI) (1). OI has been observed in patients with neurodegenerative diseases (NDDs) such as Alzheimer's disease (2), Parkinson's disease (PD) (3), or progressive supranuclear palsy (4); therefore, OI might be an early biomarker for a broad spectrum of NDDs. For example, in the neuropathological progression of PD, the olfactory bulb and the anterior olfactory nucleus are one of the first lesions within the central nervous system during the pathogenesis of PD. The lesions would continue to extend into more remote olfactory sites during the three to four pathological stages onward (5). Thus, olfactory impairment might be a manifestation and a potential diagnostic marker for early PD (6). Published literature indicates that OI is associated not only with NDDs, but also with increased mortality. A few longitudinal studies have shown an excessive relative risk for mortality as high as 20–112% in the elderly with OI, adjusted for age, sex, and other covariates (7, 8). Some studies have demonstrated the OI-mortality relationship in the middle-aged group (9, 10). In addition, some evidence indicates that the NDD is one of the leading causes of death and probably would overtake cancer by 2040 as the top one killer (11). Therefore, the NDD was considered as a possible mediator in the relationship between OI and mortality and explains the relationship partly (8, 12, 13).

However, the question arises as whether OI could proceed with the onset of neuropathological lesions or simply cause or potentiate by the neuropathologies (14)? Furthermore, cognitive performance at baseline may also tangle with the relationship (15), which makes the question even more complicated. So far, few studies have addressed the question and the findings remain controversial because of the diverse study design and data analysis method. In this study, we analyzed data collected from the prospective phase of the 10-year follow-up of the Shanghai Aging Study (SAS) to explore the mediation effect of NDDs on the OI-mortality relationship.

## Methods

### Study Participants

The SAS is a population-based prospective cohort study conducted among the older adults residing in a community of downtown Shanghai, China. The study design and recruitment process of the cohort have been described in detail elsewhere (16). A total of 1,811 participants aged 60 years or older (mean age = 70 years) who completed both an olfactory identification test and a cognitive assessment at baseline (2010–2011) were included in this study.

### Baseline Data Collection

Baseline data collection has been described in detail previously (17). Information on demographics and lifestyle of the participants including age, sex, education, smoking, and alcohol drinking were collected *via* an interviewer-administered questionnaire. The body mass index (BMI) of each participant was calculated by using his/her height and weight measured by a research nurse. Physical activity was measured in metabolic equivalent (MET) value. Information on previous and/or current chronic diseases was inquired based on the medical records. Apolipoprotein E (APOE) genotyping was conducted by using the blood or saliva samples of the participants and APOE-ε4 allele positive was defined as the presence of at least one ε4 allele.

Olfactory function was assessed by using the Sniffin' Sticks Screening Test-12 (SSST-12), which consists of 12 odors (orange, leather, cinnamon, peppermint, banana, lemon, liquorice, coffee, cloves, pineapple, rose, and fish) presenting on felt-tip sticks (18).

### Diagnosis of Neurodegenerative Diseases

At baseline, participants were invited for a clinical interview. Cognitive function was evaluated by using a battery of neuropsychological tests including the Mini-Mental State Examination (MMSE), Conflicting Instructions Task, Modified Common Object Sorting Test, Auditory Verbal Learning Test, and Renminbi (Chinese currency) Test. The normative data and detailed description of the assessment battery were reported elsewhere (19). A panel of experts reached a consensus for diagnosis of dementia and mild cognitive impairment (MCI) based on the criteria of the Diagnostic and Statistical Manual of Mental Disorders, the 4th edition, and the Peterson criteria (20, 21). PD, Huntington's disease, and other NDDs were reported by the participants and confirmed with their medical records.

### Mortality During Follow-Up

Survival statuses of the participants from baseline to December 31, 2019 were obtained from the mortality surveillance system of the local Centers for Disease Control and Prevention, which is responsible for verifying the date of death and causes of death from the death certificate (22).

### Statistical Methods

The continuous variables were presented as mean with standard deviation (SD) and the categorical variables were presented as count and percentage (%). In this study, OI was defined as the total SSST-12 score < 8 (the median of the SSST-12 scores). The Pearson's chi-squared test was used to test the differences between the OI and non- or mild OI groups for categorical variables and the Student's *t*-test and the Mann–Whitney *U* test were used for continuous variables.

**Figure 1:**
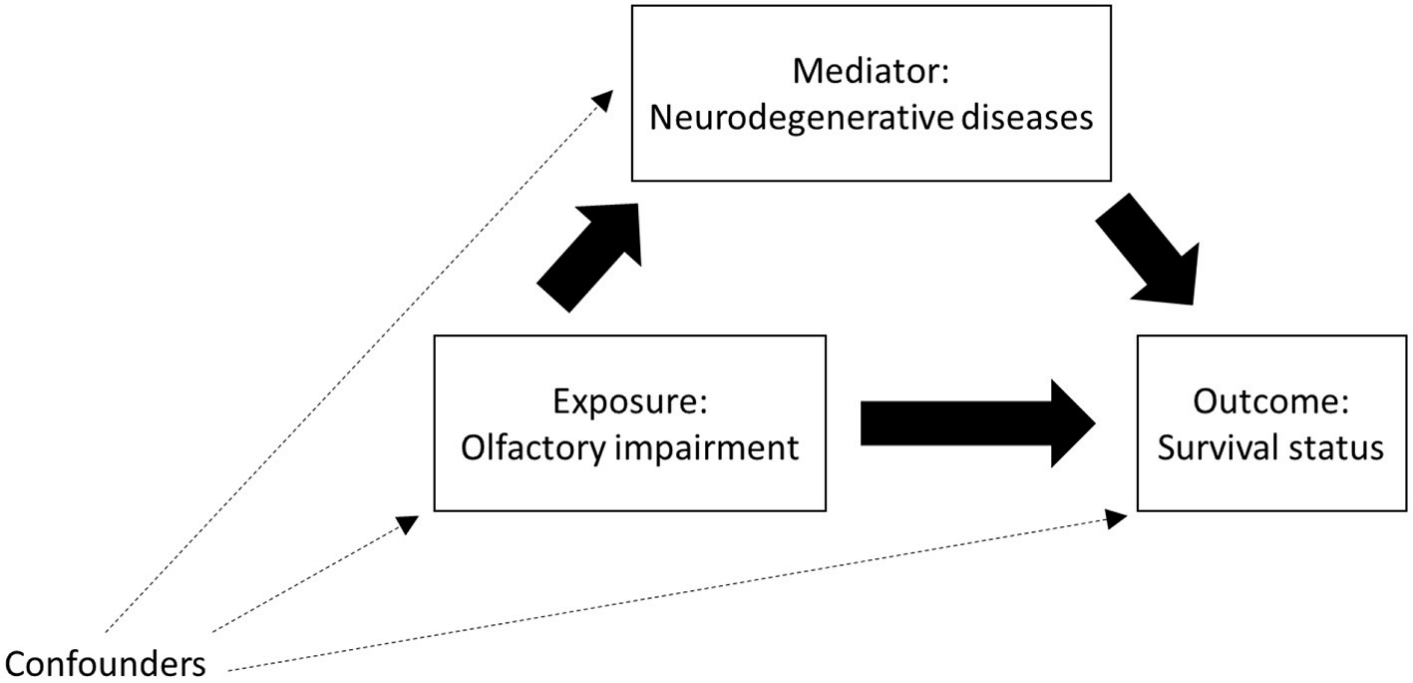
Directed acyclic graph for the association of olfactory impairment **(OI)** with mortality, mediated by neurodegenerative diseases **(NDDs)**, adjusted for the associations of potential confounders with OI, NDDs, and mortality.

The directed acyclic graph in Figure 1 illustrates the hypothetical association paths between OI and the survival status of the participants mediated by NDDs. The four-way decomposition method was used to attribute effects to interaction and mediation (23). Association between OI and death was evaluated by using the Cox proportional hazards regression model. The proportional hazards assumption was tested on the basis of Schoenfeld residuals. Association between OI and the mediator, i.e., NDDs was assessed by using the logistic regression model. To exclude the multicollinearity between the independent variables included in the logistic regression models, the bidirectional stepwise variable selection method was implemented before the mediation and interaction analysis.

**Figure 2:**
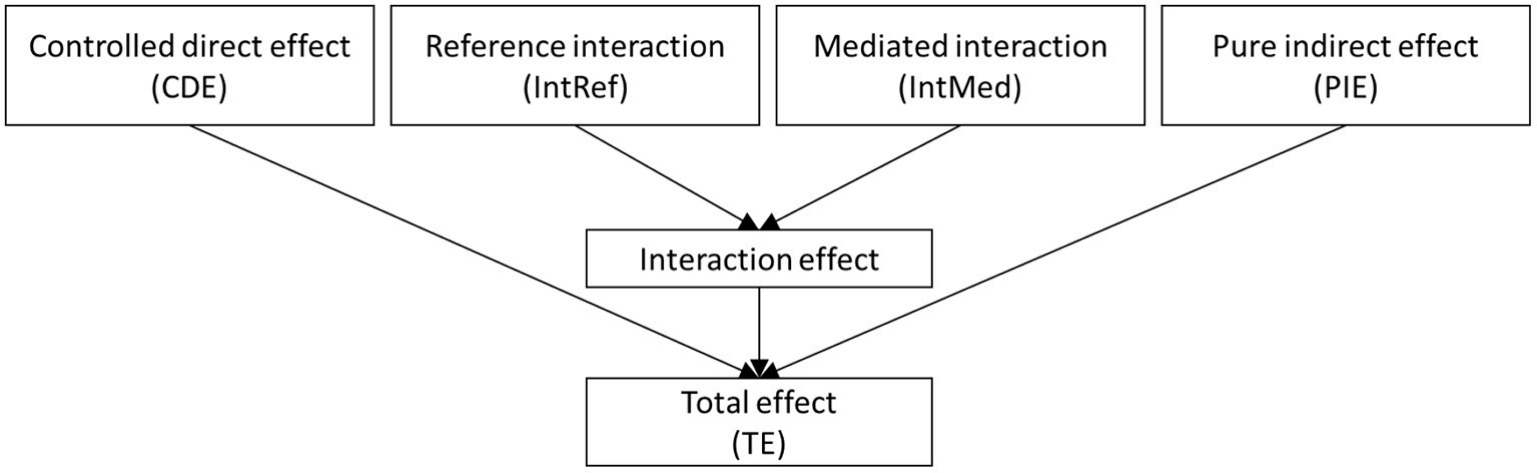
The four-way decomposition method encompasses both the components for mediation and interaction.

The total effect of OI on death in excess relative risk (ERR) scale, i.e., a hazard ratio (HR) from the Cox proportional hazards regression model minus one, was decomposed into four components: controlled directed effect (CDE) due to OI only, mediated main effect or pure indirect effect (PIE) due to mediation only, reference interaction (IntRef) due to interaction only, and mediated interaction (IntMed) due to mediation and interaction (24). The relationship of the four components is shown in Figure 2. The effect decomposition analysis was conducted for both the OI and failure to identify individual odors.

All the statistical analyses were conducted in Stata 16.1 (StataCorp LLC, College Station, Texas, USA). A two-sided *p*-value was considered as statistically significant and a 95% CI was provided for each estimated effect.

## Results

### Baseline Characteristics of the Participants and Outcomes

In the total 1,811 participants recruited in the SAS cohort, 662 participants were identified as OI at baseline, 378 participants were diagnosed as NDDs (29 dementia, 5 PD, and 344 MCI), and 258 participants died during an averagely 9.4 years of follow-up. Baseline characteristics of the participants and the outcomes during the follow-up are shown in Table 1. Compared to the non-OI group, the OI group was older, less educated, with a higher proportion of hypertension, stroke, chronic bronchitis, the lower MMSE score, higher activities of daily living (ADL) score, lower total cholesterol (TC) and low-density lipoprotein (LDL), and a higher proportion of failure to identify individual odors, NDDs, and death (Table 1).

**Table 1:** Baseline characteristics of the participants and follow-up outcomes.

**Variable**	**All**	**Non-OI**	**OI**	***p*-value**
N	1,811	1,149	662	
Female (%)	983 (54.3)	621 (54.0)	362 (54.7)	0.832
Age, years (mean ± SD)	70.22 (7.21)	68.92 (6.81)	72.47 (7.34)	< 0.001
BMI, kg/m^2^ (mean ± SD)	24.28 (3.66)	24.38 (3.73)	24.09 (3.52)	0.108
Education, years [median (IQR)]	12.00 [9.00, 15.00]	12.00 [12.00, 16.00]	12.00 [9.00, 15.00]	< 0.001
Smoking (%)	186 (10.3)	119 (10.4)	67 (10.1)	0.937
Drinking (%)	142 (7.8)	84 (7.3)	58 (8.8)	0.310
Physical activity [median (IQR)]	21.00 [9.80, 37.10]	21.00 [10.03, 37.10]	21.00 [9.80, 38.30]	0.748
NDDs (%)	378 (20.9)	163 (14.2)	215 (32.5)	< 0.001
Coronary heart disease (%)	200 (11.0)	117 (10.2)	83 (12.5)	0.144
Hypertension (%)	983 (54.3)	593 (51.6)	390 (58.9)	0.003
Diabetes (%)	251 (13.9)	153 (13.3)	98 (14.8)	0.417
Depression (%)	285 (15.7)	169 (14.7)	116 (17.5)	0.129
Stroke (%)	212 (11.7)	118 (10.3)	94 (14.2)	0.015
Cancer (%)	191 (10.5)	129 (11.2)	62 (9.4)	0.245
Chronic kidney disease (%)	108 (6.0)	63 (5.5)	45 (6.8)	0.301
Anemia (%)	24 (1.3)	16 (1.4)	8 (1.2)	0.907
Urinary tract infections (%)	574 (31.7)	352 (30.6)	222 (33.5)	0.221
Chronicbronchitis (%)	262 (14.5)	146 (12.7)	116 (17.5)	0.006
MMSE score (mean ± SD)	28.20 (2.30)	28.61 (1.69)	27.47 (2.95)	< 0.001
ADL score (mean ± SD)	20.54 (3.34)	20.26 (2.04)	21.03 (4.80)	< 0.001
TC, mmol/L (mean ± SD)	5.40 (1.05)	5.46 (1.07)	5.29 (1.00)	0.001
TG, mmol/L (mean ± SD)	1.71 (1.05)	1.75 (1.02)	1.65 (1.09)	0.067
HDL, mmol/L (mean ± SD)	1.34 (0.35)	1.34 (0.35)	1.32 (0.35)	0.242
LDL, mmol/L (mean ± SD)	3.25 (0.87)	3.29 (0.89)	3.18 (0.84)	0.011
APOEε4 positive (%)	312 (17.2)	199 (17.3)	113 (17.1)	0.971
Fail to identify odor (%)				
Orange	432 (23.9)	175 (15.2)	257 (38.8)	< 0.001
Leather	797 (44.0)	353 (30.7)	444 (67.1)	< 0.001
Cinnamon	1,034 (57.1)	530 (46.1)	504 (76.1)	< 0.001
Peppermint	172 (9.5)	34 (3.0)	138 (20.8)	< 0.001
Banana	632 (34.9)	248 (21.6)	384 (58.0)	< 0.001
Lemon	836 (46.2)	426 (37.1)	410 (61.9)	< 0.001
Liquorice	844 (46.6)	395 (34.4)	449 (67.8)	< 0.001
Coffee	150 (8.3)	12 (1.0)	138 (20.8)	< 0.001
Cloves	884 (48.8)	461 (40.1)	423 (63.9)	< 0.001
Pineapple	562 (31.0)	215 (18.7)	347 (52.4)	< 0.001
Rose	691 (38.2)	276 (24.0)	415 (62.7)	< 0.001
Fish	322 (17.8)	95 (8.3)	227 (34.3)	< 0.001
Death (%)	258 (14.2)	115 (10.0)	143 (21.6)	< 0.001

*Physical activity: in metabolic equivalent (MET) value*.

*BMI, body mass index; NDDs, neurodegenerative diseases; MMSE, mini-mental state examination; ADL, activities of daily living; TC, total cholesterol; TG, triglyceride; HDL, high-density lipoprotein; LDL, low-density lipoprotein; APOE, Apolipoprotein E*.

### Association Between OI and Mortality Mediated by NDDs

No statistically significant association was found between OI and death in the general Cox proportional hazards regression model (HR = 1.25, 95% CI: 0.90–1.72, *p* = 0.181); however, OI was statistically significantly associated with ND [odds ratio (OR) = 2.30, 95% CI: 1.80–2.94, *p* < 0.001] (Table 2). The four-way decomposition method revealed a statistically significant association of OI with death. Overall, 43% higher risk for death was associated with OI (ERR = 0.43, 95% CI: 0.06–0.80, *p* = 0.023). Excluding the mediation from NDDs and interaction between OI and NDDs, the controlled direct effect of OI on death was even higher in NDDs participants, with an ERR of 77% (95% CI: 0.00–1.55, *p* = 0.050) (Table 2).

**Table 2:** Results of the four-way decomposition analysis for the association between OI and mortality.

	**Estimates**	**95% CI**	***p*-value**
Model for outcome (Cox regression, HR^*^)			
OI	1.25	0.90–1.72	0.181
NDDs	1.35	0.87–2.12	0.178
OI × NDDs	1.28	0.73–2.25	0.387
Model for mediator (logistic regression, OR^*^)			
OI	2.30	1.80–2.94	< 0.001
Decomposition of excess relative risk (ERR)^*^			
TE(olfactory-death)	0.43	0.06–0.80	0.023
CDE			
NDDs	0.77	0.00–1.55	0.050
Non NDDs	0.23	−0.14–0.61	0.223
IntRef			
NDDs	−0.46	−1.15–0.23	0.193
Non NDDs	0.08	−0.04–0.20	0.195
IntMed	0.07	−0.04–0.19	0.202
PIE	0.05	−0.03–0.12	0.231

* *Adjusted for sex, age, coronary heart disease, ADL, anemia, LDL, and chronic kidney disease*.

*ADL, higher activities of daily living; LDL, low-density lipoprotein. HR, hazard ratio; OI, olfactory impairment; NDDs, neurodegenerative diseases; TE, total effect; CDE, controlled directed effect; IntRef, reference interaction; IntMed, mediated interaction ; PIE, pure indirect effect*.

### Association Between Failure to Identify Individual Odors and Mortality Mediated by NDDs

**Figure 3:**
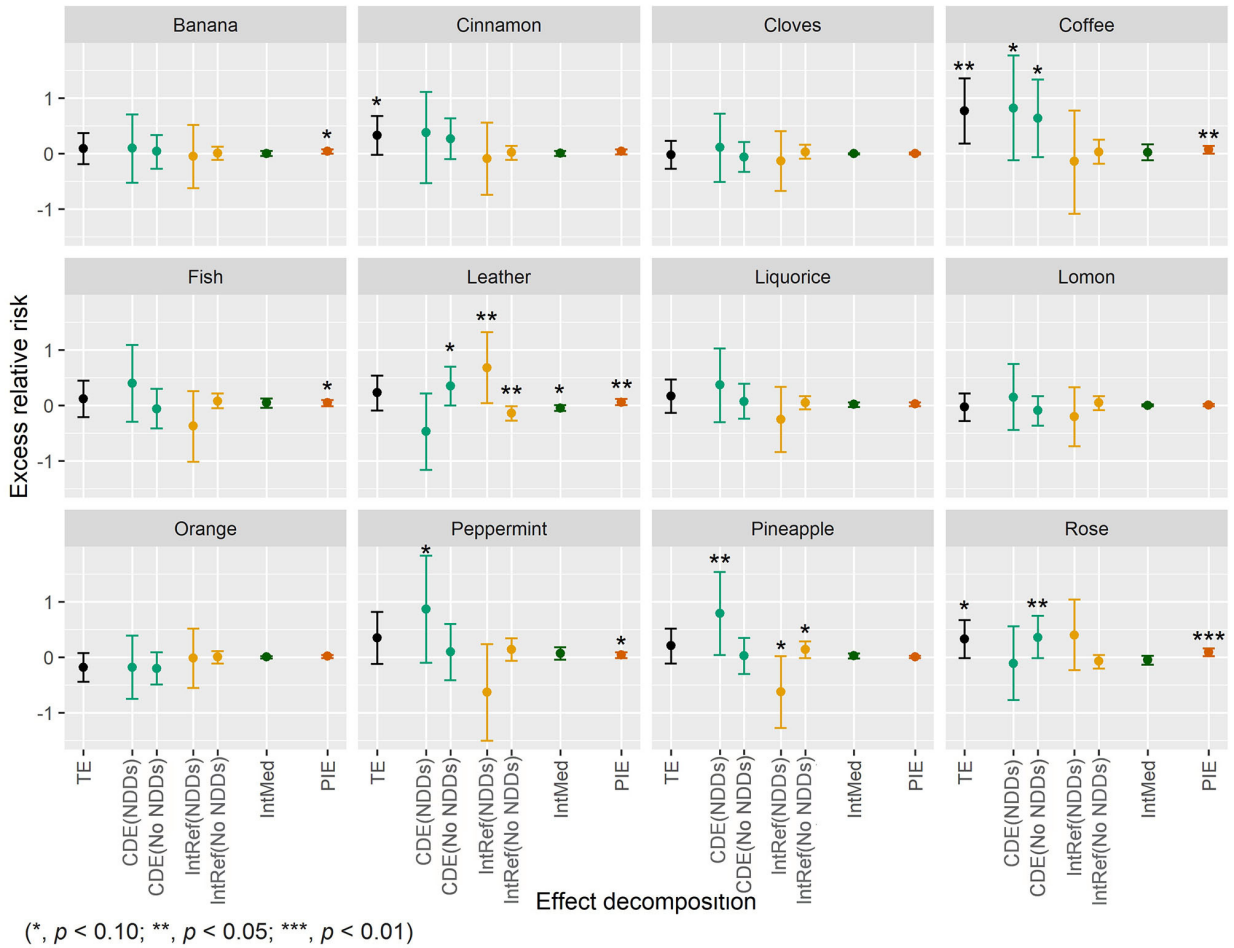
The four-way decomposition method of excess relative risk of mortality for individual odors.

Statistically significant association was found for failure to identify coffee (ERR = 0.77, 95% CI: 0.18–1.36, *p* = 0.010) and marginally significant associations were found for failure to identify cinnamon (ERR = 0.33, 95% CI: −0.02–0.68, *p* = 0.068) and rose (ERR = 0.33, 95% CI: −0.01–0.67, *p* = 0.054) with death (Figure 3). No statistically significant interaction was found between failure to identify the three individual odors and NDDs. Interestingly, statistically significant mediation by NDDs was found for failure to identify coffee and rose with PIEs of 0.07 (95% CI: 0.00–0.14, *p* = 0.039) and 0.09 (95% CI: 0.02–0.16, *p* = 0.016), respectively (Figure 3). However, the mediation from NDDs only contributed to a small part (9.1%) of the association of failure to identify coffee with death, while a considerable mediation (27.2% of total ERR) from NDDs was found in the association between failure to identify rose and death. Besides, the controlled direct effect of failure to identify coffee on death was statistically marginally significant, with ERRs of 0.82 (95% CI: −0.12–1.77, *p* = 0.089) and 0.64 (95% CI: −0.06–1.34, *p* = 0.071) for NDDs and non-NDDs participants, respectively. No statistically significant association was found for failure to identify any other odor and death (Figure 3).

## Discussion

Olfactory dysfunction is reported as the only sensory, which has been associated with mortality, when compared with hearing or visual impairment (2). Although the OI-mortality relationship has been identified independently from incidence of dementia (7), current findings support the relationships between OI, NDDs, and mortality risk and some studies have further shown that cognitive function might be a potential mediator in the relationship (3, 12, 13). Devanand et al. found that the OI-mortality association was weaker when controlled for dementia, yet still statistically significant (13). Liu et al. showed that dementia could account for 22% of the higher 10-year mortality linked to poor olfactory function (3). Our findings have confirmed that OI was associated with mortality in older adults during a 9.4-year follow-up and the association was even stronger in the NDDs participants.

We also found that failure to identify certain odors, especially coffee or rose, was associated with a higher mortality risk and the association was mediated by NDDs. Failure to identify a specific odor-like coffee was also suggestive of reduced survival in another study (25); however, failure to smell rose has not been reported for this potential connection. The mediation of NDDs on the associations of coffee is weak. One hypothetical explanation for the absence or weak mediation of NDDs might be that OI happened, while the clinical cognitive function is not yet affected and, thus, undiagnosed (26); postmortem markers of NDDs existing in the brains of subjects without a diagnosis of NDDs have supported this speculation (27).

There are several strengths in this study. First, although a causal relationship between OI and mortality and the mediation by NDDs cannot be established in the observational study, the study design and the sequential happening of the exposure, mediator, and outcome have ruled out a reversed conclusion. Second, cognitive function at baseline has been controlled in this study. Third, abundant demographic and medical historical variables collected in the SAS offered us a unique opportunity to use the four-way decomposition method to attribute effects to interaction and to assess mediation. The four-way decomposition methods used in published related literature were essentially special cases of the four-way decomposition. The four-way decomposition method could provide maximum insight into how much of an effect is mediated by a potential mediator in an exposure-mediation-outcome causal pathway (23).

Limitations of this study cannot be ignored. First, besides the complicated diagnosis procedure of cognitive impairment, we defined participants with other NDDs only based on their medical records, not used standard inclusion/exclusion criteria. Some participants might have NDDs, but they ignored that and did not go to see doctors. So, the NDDs in this study might be underestimated. On the other hand, there may be misdiagnosis existed because NDDs diagnoses on the medical records were made by doctors from different levels of hospitals. Second, NDDs include a large spectrum of disorders with remarkable differences. In this study, it is not appropriate to take dementia, MCI, and PD into account as the homogeneous NDD. Third, the instrument we used to assess the olfactory function only has 12 sticks for 12 odors. This limited us in seeking odors that were more sensitive than these 12. Fourth, the SSST-12 evaluated the olfactory identification, which cannot completely present the olfactory function. Together with the odor discrimination, detection thresholds should be administered to define the olfactory impairment. Fifth, this study was an observational study with relatively small sample size. The association and the mediation effects resulting from the statistical analysis only could reflect the phenomenon or clue. Future studies should focus on the biological mechanism, which could deeply explain our findings.

## Conclusion

This study indicated that OI was associated with the long-term mortality in older adults and the association was even stronger in those with NDDs. Failure to identify coffee or rose was associated with a higher mortality risk and the association was mediated by NDDs. This study result suggests a potential signal for predicting survival in the elderly, especially those with NDDs. The findings need to be further confirmed with larger cohort studies or with interventional design.

## Data Availability Statement

The raw data supporting the conclusions of this article will be made available by the authors, without undue reservation.

## Ethics Statement

The studies involving human participants were reviewed and approved by the Shanghai Aging Study was approved by the Medical Ethical Committee of Huashan Hospital, Fudan University, Shanghai, China (approval number: 2009-195). The patients/participants provided their written informed consent to participate in this study.

## Author Contributions

YC and DD contributed to the design of the study, data analysis and interpretation, and drafting of the manuscript. ZX contributed drafting of the manuscript and data analysis. ZX, WW, and QZ contributed to the data collection of the study. All authors contributed to the critical revision of the manuscript for important intellectual content.

## Funding

DD and QZ were supported by grants from the Shanghai Municipal Science and Technology Major Project (No. 2018SHZDZX01), the ZJ Lab, National Natural Science Foundation of China (81773513), and the State Key Laboratory of Neurobiology and Frontiers Center for Brain Science of Ministry of Education, Fudan University. The funding agencies had no role in the design of this study and will not have any role during its execution, analyses, interpretation of the data, or decision to submit results.

## Conflict of Interest

The authors declare that the research was conducted in the absence of any commercial or financial relationships that could be construed as a potential conflict of interest.

## Publisher's Note

All claims expressed in this article are solely those of the authors and do not necessarily represent those of their affiliated organizations, or those of the publisher, the editors and the reviewers. Any product that may be evaluated in this article, or claim that may be made by its manufacturer, is not guaranteed or endorsed by the publisher.
